# Epiemiologic Features and Hospitalization Cost of Burn Injuries in Iran Based on National Burn Registry; a Cross-sectional Study

**Published:** 2019-11-02

**Authors:** Reza Rezaee, Khalil Alimohamadzadeh, Seyed-Mojtaba Hossini

**Affiliations:** 1 Department of Health Service Management, North Tehran Branch of IslamicAzad University, Tehran, Iran.

**Keywords:** Burns, costs and cost analysis, epidemiology, accident prevention

## Abstract

**Introduction:**

Burn is one of the most common causes of injury in the world. The aim of this study was to determine the epidemiologic features and cost of hospitalization associated with burn injuries in Iran.

**Methods:**

In this cross-sectional study, the data related to hospitalized burn cases in 2017 were obtained from the office of curative affairs in the Ministry of Health and Medical Education of Iran and analyzed regarding the epidemiologic features and hospitalization costs.

**Results:**

35933 hospitalized burn patients, from the beginning to the end of 2017, with the mean age of 29.37 ± 21.41 (1 – 99) years were studied (59.4% male). Scald burns (49.4%) were the most prevalent type of burns and 30.3% of burns occurred in spring. The most frequent severity of burn injury was second-degree burns (69.3%) with 1-20 percent involvement of body's surface area (74.7%). Frequency of scald burn was higher in females, while the frequency of flame was higher in males. Total hospitalization cost of studied cases was 66910.22 $. In male patients, the highest average direct cost was related to electrical burns; while in females, the highest average direct cost was related to chemical burns. Higher degrees and percentages of burn injuries required a more costly treatment.

**Conclusions:**

Burn injuries most frequently happened in males, ages < 10 years, spring season, and with scald and flame. The most frequent injuries were second-degree burns with 1% – 20% body surface involvement. The highest direct hospitalization cost was related to chemical and electrical burns. There was a direct correlation between the degree and percentage of burn and the hospitalization costs.

## Introduction

Burn injuries and their related outcomes are major causes of mortality and disability in Iran and all over the world (1). Globally, about 195,000 deaths per year are attributed to burn injuries (2). Burn care is generally considered an expensive care (3) and this statement is clearly expressed in some recent studies (4-7). In Iran, burn injuries have ranked thirteenth in the burden of disease. They are among the top twenty outcomes with the highest rates of mortality and disability (8). In general, most burns occur in developing countries with low or medium incomes (9) with more than half of them happening in Asia (10). The extent, severity, and degree of burns are the most important predictors of death in burn injuries (11). Various risk factors of mortality have been reported in different studies such as age under 4 and over 65 years (12, 13), male gender (14), poverty (15), crowding (16) and so on. Awareness of socioeconomic differences in the epidemiology of burning and recognition of etiological patterns of burn injuries could be useful in planning and adopting prevention strategies (17). The aim of this study was to determine the epidemiologic features and hospitalization cost of burn injuries in Iran. 

## Methods

***Study design and setting***

This cross-sectional study was conducted on data of all burn patients that attended burn wards of hospitals affiliated to any of the Universities of Medical Sciences in Iran, throughout 2017. The study protocol was approved by the local Human Subject Review Board in Islamic Azad University, North Tehran Branch.

***Participants***

All burn patients whose data were collected in the burn registery database throughout 2017 were evaluated. There was not any gender or age limitation.

***Data gathering***

Data were obtained as a Microsoft Excel file, from data registery of burn patients in office of curative affairs, Ministry of Health and Medical Education. All physicians in charge of burn patients were trained for filling out the related data forms. Data were compiled and checked for duplicate entries at the burn units in each hospital and were then sent to the deputy of curative affairs on a monthly basis. Data included age, gender, reporting medical university, duration of hospitalization, season, burn severity (degree and percentage), cause, and the total cost of each burn injury. 

***Statistical analysis***

All the statistical analyses were conducted using SPSS, version 21 (IBM Inc., Armonk, NY, USA). Qualitative variables were presented as absolute frequency and percentage. Quantitative measures were expressed using mean and standard deviation. We used the t-test and analysis of variance (ANOVA) to compare the mean values of various continuous variables in different groups/categories. Chi-square test was used to compare categorical data. P values less than 0.05 were regarded as statistically significant.

## Results

35933 hospitalized burn patients, from the beginning to the end of 2017, with the mean age of 29.37 ± 21.41 (1 – 99) years were studied (59.4% male). Baseline characteristics of studied cases are presented in table 1. Scald burns (49.4%) were the most prevalent type of burns and 30.3% of burns occurred in spring. The most frequent severity of burn injury was second-degree burns (69.3%) with 1-20 percent involvement of body's surface area (74.7%). 

**Figure 1:**
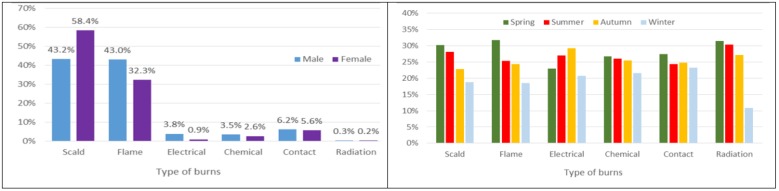
Distribution of burn injuries based on gender and season

Analyses showed that the absolute number and percentage of scald burn during the study period was higher in females [8515 (58.4%) versus 9234 (43.2%); figure 1]; while the absolute number and percentage of flame burn in males was higher than females [9188 (43.0%) versus 4702(32.3%)]. Burns due to any of the studied causes had happened most frequently in spring (30.3%) and least frequently in winter (19.1%), except for electrical burns, which had happened most frequently in autumn. 

Total hospitalization cost of studied cases was 66910.22 $. Table 2 shows the distribution of hospitalization costs associated with burns according to gender in Iran in 2017. In males, the highest average direct cost was related to electrical burns; while in females, the highest average direct cost was related to chemical burns. Also, a direct relationship was observed between the degree and percentage of burn and the costs associated with treating patients in both genders. In other words, higher degrees and percentages of burns required a more costly treatment. 

## Discussion

Based on the findings of the present study, burn injuries most frequently happened in males, ages < 10 years, spring season, and with scald and flame. The most frequent injuries were second-degree burns with 1% – 20% body surface involvement. The highest direct hospitalization cost was related to chemical and electrical burns. There was a direct correlation between the degree and percentage of burn and the hospitalization costs.

Burn injury is an important cause of hospital admission (18). Previous studies conducted in USA (19), Iran (20), and Portugal (21) concluded that burn injury is a significant source of morbidity and mortality in children. The results of the present study demonstrated that the incidence of burns was higher in children under the age of 10 years compared to other age groups, which is in line with the findings of previous studies (22, 23). Males were more affected by burns than females. The Predominant involvement of males in burn injuries was also reported by other published studies (24, 25). Low average age in our study is due to a higher incidence of accidents in boys, especially in the school-age period. Our findings regarding the most frequent type and season of burns were in line with some previous studies (22, 26). Scald [49.4 %, p<0.001] and flame [38.7 %, p<0.001] were predominant causes of burns among all patients (both genders) hospitalized due to burn injuries. We found that a high percentage of burns over the course of the present study (except electrical burns) occurred in the spring season, while the frequency of these causes decreased in the winter season.

**Table 1:** Baseline characteristics of studied burn cases

**Characteristics**	**Number (%)**
**Gender**	
Male	21354 (59.4)
Female	14575 (40.6)
**Age (year)**	
<9	9321 (25.9)
10-19	3572 (9.9)
20-29	63.52 (17.7)
30-39	6588 (18.3)
40-49	3833 (10.7)
50-59	2863 (8.0)
≥60	3404 (9.5)
**Season**	
Spring	10901 (30.3)
Summer	9597 (26.7)
Autumn	8571 (23.9)
Winter	6864 (19.1)
**Type of burns**	
Scald	17749 (49.4)
Flame	13890 (38.7)
Electrical	936 (2.6)
Chemical	1120 (3.1)
Contact	2146 (6.0)
Radiation	92 (0.3)
**Degree of burns**	
Burn I	2121 (5.9)
Burn II	21912 (69.3)
Burn III	8634 (24.0)
Burn IV	266 (0.7)
**Body's surface (%)**	
1-10	16.27 (44.6)
11-20	10809 (30.1)
21-30	2961 (8.2)
31-40	3135 (8.7)
41-50	1289 (3.6)
>50	1712 (4.8)

**Table 2:** Hospitalization cost of burn injuries in Iran, throughout 2017, based on different baseline variables

**Variable**	**Total **	**Male**	**Female**	** P**
		Direct cost* (%)	**Direct cost (%)**	
**Age (years)**				
<9	9040.55	6559.32 (18.83)	2481.23 (7.74)	0.001
10-19	5885.46	2591.18 (7.44)	3294.28 (10.27)	0.001
20-29	5879.94	2863.95 (8.22)	3015.99 (9.40)	0.209
30-39	8268.35	3945.34 (11.33)	4323.01 (13.48)	0.011
40-49	12115.43	5908.98 (16.96)	6206.45 (19.35)	0.234
50-59	14493.73	8193.67 (23.52)	6300.06 (19.64)	0.001
≥60	11226.76	4770.35 (13.69)	6456.41 (20.13)	0.001
**Type of burns**				
Scald	7928.54	4407.12 (13.34)	3521.42 (4.57)	0.001
Flame	9686.11	4991.39 (15.11)	4694.72 (6.10)	0.019
Electrical	60510.55	6541.89 (19.81)	53968.66 (70.11)	0.097
Chemical	13811.43	6219.64 (18.83)	7591.79 (9.86)	0.003
Contact	9554.34	5447.21 (16.49)	4107.13 (5.34)	0.001
Radiation	8518.29	5420.71 (16.41)	3097.58 (4.02)	0.094
**Degree of burns**				
Burn I	5685.83	4093.76 (13.38)	1592.07 (5.33)	0.001
Burn II	7319	4176.13 (13.65)	3142.87 (10.52)	0.001
Burn III	13657.8	6666.17 (21.78)	6991.63 (23.40)	0.035
Burn IV	33826.95	15668.70 (51.20	18158.25 (60.76)	0.017
**Body's surface (%)**				
1-10	4604.89	2659.17 (6.25)	1945.72 (4.64)	0.001
11-20	11093.89	6440.27 (15.14)	4653.62 (11.10)	0.001
21-30	4121.14	2021.15 (4.75)	2099.99 (5.01)	0.487
31-40	9336.35	4446.86 (10.45)	4889.49 (11.67)	0.003
41-50	19390.56	9863.61 (23.19)	9526.95 (22.73)	0.134
>50	35897.7	17104.72 (40.21)	18792.98 (44.84)	0.001
**Total**	321853.6	141001.29	174552.2	

* Mean direct costs in US dollars (% of total cost).

 Contradictions exist between various studies regarding the distribution of leading causes of burns. These differences are mostly due to the differences in study method, the society under study, cultural and economic differences, lifestyle, and geographic location. Our results revealed that electrical burns had the highest frequency in males, while chemical burns were the most common type of burns among females. We also observed a direct positive relationship between the degree and percentage of burn increases and costs associated with treating patients. 

We believe that this evidence should be taken into account to prevent and reduce the burden of burn injuries. In summary, our findings in the current study can contribute to development of a prevention program to protect the population from burn injuries and the findings can also help prepare public and specific targeted interventions to promote safe behavior.

## Limitations

Some limitations were recognized in our study. Given that the data from this study were extracted from the Microsoft EXCEL sheet provided by curative affairs associated with Ministry of Health and Medical Education of Iran in 2017, there were no details about the type of liquids or other sources or causes of burns. Also, there was no information about the final outcome (death or survival) of patients with a burn.

## Conclusion:

Based on the findings of the present study, burn injuries most frequently happened in males, ages < 10 years, spring season, and with scald and flame. The most frequent injuries were second-degree burns with 1% – 20% body surface involvement. The highest direct hospitalization cost was related to chemical and electrical burns. There was a direct correlation between the degree and percentage of burn and hospitalization costs.
